# Microbial Quality and Phylogenetic Diversity of Fresh Rainwater and Tropical Freshwater Reservoir

**DOI:** 10.1371/journal.pone.0100737

**Published:** 2014-06-30

**Authors:** Rajni Kaushik, Rajasekhar Balasubramanian, Hugh Dunstan

**Affiliations:** 1 Singapore-Delft Water Alliance, National University of Singapore, Singapore, Singapore; 2 Department of Civil and Environmental Engineering, National University of Singapore, Singapore, Singapore; 3 School of Environmental and Life Sciences, The University of Newcastle, Callahan, NSW, Australia; U. S. Salinity Lab, United States of America

## Abstract

The impact of rainwater on the microbial quality of a tropical freshwater reservoir through atmospheric wet deposition of microorganisms was studied for the first time. Reservoir water samples were collected at four different sampling points and rainwater samples were collected in the immediate vicinity of the reservoir sites for a period of four months (January to April, 2012) during the Northeast monsoon period. Microbial quality of all fresh rainwater and reservoir water samples was assessed based on the counts for the microbial indicators: *Escherichia coli (E. coli)*, total coliforms, and *Enterococci* along with total heterotrophic plate counts (HPC). The taxonomic richness and phylogenetic relationship of the freshwater reservoir with those of the fresh rainwater were also assessed using 16 S rRNA gene clone library construction. The levels of *E. coli* were found to be in the range of 0 CFU/100 mL – 75 CFU/100 mL for the rainwater, and were 10–94 CFU/100 mL for the reservoir water. The sampling sites that were influenced by highway traffic emissions showed the maximum counts for all the bacterial indicators assessed. There was no significant increase in the bacterial abundances observed in the reservoir water immediately following rainfall. However, the composite fresh rainwater and reservoir water samples exhibited broad phylogenetic diversity, including sequences representing *Betaproteobacteria*, *Alphaproteobacteria*, *Gammaproteobacteria, Actinobacteria, Lentisphaerae a*nd *Bacteriodetes*. Members of the *Betaproteobacteria* group were the most dominant in both fresh rainwater and reservoir water, followed by *Alphaproteobacteria, Sphingobacteria, Actinobacteria* and *Gammaproteobacteria*.

## Introduction

Airborne microorgansims can be transferred to aquatic systems through atmospheric fallout of coarse particles (dry deposition) [1] and rainfall (wet deposition) [2]–[3], leading to changes in the microbial composition of receiving water bodies [4]–[5]. The presence of bacterial pathogens in airborne particulate matter (PM) is of particular concern from the public health perspective as these aerosolized bacteria can form new cells in PM [6] and be metabolically active with the potential to biogeochemically mediate atmospheric chemistry [7]–[9]. The transfer of PM containing viable bacterial pathogens from the atmosphere to water bodies can affect not only ecotoxicology, but also human health through various exposure pathways.

Pathogenic episodes in lakes and reservoirs are often associated with rain events and riverine inflows [4], [10]. Excessive rainfall has been reported to be a significant contributor to historical waterborne disease outbreaks due to mobilization and transport of bacterial pathogens [10]–[12]. Stormwater runoff is a major cause of deterioration of surface water quality in urban areas. When rainfall occurs on paved surfaces, large volumes of water are swiftly carried to drains and discharged into receiving surface waters. Thus, the transport of microbial pollution from rain runoff to lakes and reservoirs is a major concern for management of natural waters worldwide [13]. Changes in the abundance of heterotrophic and coliform bacteria resident in stored water bodies have recently been reported in relation to incoming bacterial loads following rain events [5]. Hence, it is very important to understand the impact of rainfall carrying the live microbial aerosols on the quality of freshwater reservoirs in terms of changes in the abundance of pathogens, the microbial community composition and diversity.

Water treatment organizations and environmental protection agencies frequently use the presence of bacterial indicator organisms (e.g. fecal coliforms and enterococci) and their abundance in surface waters as a surrogate for the risk of contamination by actual pathogenic microorganisms [14]–[16]. Most of the studies reported in the literature characterized some bacteria residing in bulk water at various points in the drinking water supply system using cultivation-based approaches (14,17–18]. In general, heterotrophic plate counts (HPC) are used to assess the overall bacterial quality of drinking water, or natural waters [19]. Furthermore, most of the bacterial cells in natural communities are present in a viable but nonculturable (VBNC) state and therefore are non-culturable by current cultivation methods [20]–[21].

The real composition and dynamics of bacterial communities in freshwater resources remain largely unknown. In particular, limited studies have been conducted to determine the levels of bacterial pathogens in fresh rainwater prior to its collection and storage in order to assess its possible impact on the quality of roof-harvested rainwater and also that of aquatic systems upon deposition [1], [9]. The microbial quality of roof harvested rainwater is not only influenced by that of fresh rainwater, but also by the types of rooftop surfaces, the level of water tanks cleanliness, and the presence of insects' and birds' feces. However, no systematic study has been reported in the literature so far on the phylogenetic diversity of fresh rainwater describing the taxonomic richness of readily culturable organisms present in this water medium although such studies have been conducted for roof-harvested rainwater [22]. Therefore, there is a strong need for quantifying the levels of bacterial pathogens and phylogenetic diversity in both fresh rainwater and reservoir water following their simultaneous collection at the same sampling site of the reservoir.

Molecular approaches-based methods are good tools to elucidate composition of microbial communities residing in various aquatic environments [23]–[24]. These approaches exploit the use of rRNA as taxonomic marker for microorganisms [25]–[26]. The 16 S rRNA gene is tailor-made for microbial identification due to its universal presence in bacteria, extreme species sequence conservation and evolution-induced interspecies variability [27]. With the recent development and application of large-scale high throughput pyrosequencing-based method [28], community-wide spatial and temporal information on microbial community functional structure and potential activity can be rapidly obtained. Although the pyrosequencing-based approach is able to identify new sequences, it suffers from very high sensitivity to random sampling errors, dominant populations and contaminated non-target DNA [29]. The 16 S rRNA gene clone library method is a relatively old method that has been widely used in bacterial community analyses in various environments [30]–[32]. This method has been successfully applied to understand the role of freshwater bacterioplankton in global biogeochemical processes in the aquatic ecosystems [33]–[34]. However, the 16 S rRNA application to fresh rainwater has not been reported to date. The study of phylogenetic diversity in fresh rainwater and reservoir water could provide a basic understanding of the complex communities of environmental bacteria present in fresh rainwater and their impact on the composition of microbial communities in reservoir water intended for human consumption. The objectives of this work were to (1) study the impact of microbial loading of fresh rainwater on the water quality of a tropical reservoir in Singapore (characterized by heavy rainfall) upon deposition using total bacteria and traditional bacterial indicators and (2) examine the kind of bacterial diversity that fresh rainwater and reservoir water harbor using traditional 16 s rRNA cloning and sequencing method.

## Materials and Methods

### Sampling

Singapore's climate is characterized by uniform temperature and pressure, high humidity, and abundant rainfall. Singapore receives an annual rainfall of about 2400 mm. There are no distinct wet, or dry seasons as rainfall occurs every month of the year. The two main seasons, based on the prevailing dominant winds, are the Northeast monsoon season (from late November to March), and the Southwest monsoon season (from late May to September). April to early May and October to early November are generally the transitional months separating the monsoons. December is usually the wettest month with an average rainfall of 280 mm.

**Figure 1:**
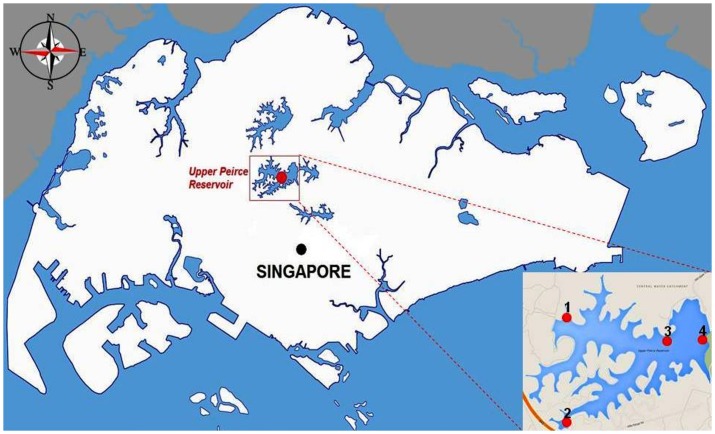
Outline of the four sampling sites at the tropical reservoir of Singapore used in this study.

Both rainwater samples and reservoir samples were collected at a tropical reservoir (coordinates-1°22′03″N 103°48′07″E) as part of the Singapore-Delft Water Alliance project. There was no need for any special permission to carry out the water sampling, and the activities did not involve endangered or protected species. Reservoir water samples were collected from four different sites at the reservoir using a grab sampling method along with rainwater samples. Both types of water samples were collected concurrently at the same four sites (No. 1, 2, 3 and 4). The reservoir has a capacity of 27.8 million of water over 304 hectares of water surface. Site 1 was located at the centre of the reservoir with no land use. Site 2 was located at the corner of the reservoir with influences from a local drainage system. Site 3 was situated near a major highway and site 4 was near a golf course, having anthropogenic influence from this land use type (Fig.1).

Both rainwater and reservoir water samples were collected from the sampling locations at regular weekly intervals and within 24 h of rainfall events between January 2012 and April 2012. A total of 33 rain samples on an event-to-event basis were collected (n = 6, 5, 12, and 10 for January, February, March and April, respectively). On each sampling occasion two replicate grab samples (1 L) were collected 1 m from the shore at a depth of ∼0.5 m in sterile containers. 1 Liter of fresh rainwater samples was collected in sterilized glass bottles (amber colored glass bottles with a sterilized funnel attached to them) placed at all the four sites used for reservoir sampling. Both fresh rainwater and reservoir water samples (immediately after rainfall) collected from all the four sites were transferred to 2 L sterilized bottles for microbiological and chemical analyses. These water samples were transported to the laboratory in a chilled-cold box and processed within 6 h of collection. The samples were first analyzed for physico-chemical analyses; pH, conductivity, turbidity and conductivity were measured for all the samples as provided in Text S1,Table S1.

### Microbiological Analyses

Concentrations of total coliform bacteria and *E. coli* were determined using the m-ColiBlue24 membrane filtration system (Millipore, Cat #M00PMCB24, Bedford, Massachusetts). For all the collected water samples, 100 mL of the sample was filtered onto cellulose ester membranes using vacuum filtration and the membrane was then incubated for 24 h in sterile petri dishes containing absorbent pads soaked with 2 mL of m-ColiBlue24 broth at 37°C. This was performed in duplicate for all the samples. The colonies in blue color were indicative of *E. coli*, while total coliforms were enumerated by counting the colonies in red color. The average CFU/100 mL values obtained for rain events in each month for *E. coli* and total coliforms were estimated.

HPC and *Enterococci* counts were also determined based on average counts obtained for all the samples collected at the four sites in this study. Briefly, for HPC enumeration, one mL of each water sample was serially diluted and the dilutions were aseptically plated in duplicates onto a plate count agar (Sigma-Aldrich, USA) and incubated at 37°C for a maximum duration of 48 h. The average colony counts were expressed as CFU/mL.

For *Enterococci*, the enumeration was performed as per the USEPA Method [35]. In brief, 100 mL of the water sample was filtered onto cellulose esters membranes using vacuum filtration in duplicate and the membranes were then placed on top of the membrane-*Enterococcus* Indoxyl-β-D-Glucoside Agar (mEI Agar, BD, NJ, USA) incubated for 24 h at 41±0.5°C. Colonies with a blue halo, regardless of color, were enumerated as *Enterococci*. The colony counts were expressed as average CFU/100 mL.

### DNA Extraction

One liter of each fresh rainwater and reservoir water samples (duplicates for each sampling site and date) was first filtered through a pre-combusted glass fiber filter (90-mm diameter, GF/F, Whatman) followed by a 0.22 µm hydrophilic polycarbonate filter (Millipore Corporation, Bedford, MA). The filters were cut into small pieces and suspended in 1.0 mL of phosphate buffered saline and DNA was extracted using Ultraclean Microbial DNA Isolation kits (MO BIO laboratories, Carlsbad, CA) according to the manufacturer's instructions. The extracted DNA solution was stored at −80°C.

### Construction of 16 S rRNA gene libraries

Clone libraries were constructed from composite DNA samples of fresh rainwater and composite DNA samples of reservoir water obtained at the four sites. Briefly, the 16 S rRNA gene was amplified from pooled environmental DNA samples using primers 27f (5′-AGAGTTTGATCMTGGCTCAG-3′) and 1492r (5′-TACCTTGTTACGACTT-3′) [36]. The PCR products were purified using UltraClean PCR Clean-up DNA purification kits (MO BIO Laboratories) and made up to 30 µL with water. Amplified fragments were cloned into TOPO-TA plasmids using the TOPO-TA cloning kit (Invitrogen, Carlsbad, CA) and transferred into *Escherichia coli* DH5a cells (TakaraBio, Otsu, Japan) to construct 16 S rRNA gene libraries.

Cloned plasmid inserts were amplified directly from cells as described [37] using vector primers. The 16 S rRNA gene portion of the cloned DNA was initially sequenced using the ABI Prism BigDye terminator v3.1 and cycle sequencing kit (PE Applied Biosystems). We sequenced 150 clones for each bacterial gene library.

### Phylogenetic analyses

Cloned gene sequences were vector-trimmed and aligned using the NAST (Nearest Alignment Space Termination) algorithm for creating multiple sequence alignments [38]. NAST aligned sequences were chimera checked with chimera slayer. All bioinformatics processes were performed in the MOTHUR environment [39]. Classification of sequences was done using Bayesian classifier method implemented in MOTHUR against Greengenes database [40]. The division level groupings were determined by taxonomic assignment performed by the Ribosomal Database Project 10.0 Classifier tool [41]. Pairwise distances of the aligned clone sequences were calculated and Operational Taxonomic units (OTUs) were grouped using the average neighbor method with a cut-off of 0.03 sequence similarity. Coverage of clone libraries was calculated according to the method recommended by Good (1953) [42]. A Phylogenetic tree was constructed using the neighbor- joining method of ARB with Jukes-Cantor correction model and with 1000 bootstrap replications [43].

### Community Statistical Analyses

Microbial quality data were subjected to the student t-test for determining statistical significance. For microbial diversity, analyses of beta diversity with replicate data from rainwater and its respective reservoir water were used for comparison of the two microbial communities. All profiles were inter-compared in a pair-wise fashion to determine a dissimilarity score and stored in a distance matrix. The Unifrac distance metric, as described in Lozupone et al. 2006, was utilized for the phylogenetic distance between OTUs to determine the dissimilarity between the two communities [44]. Weighted Unifrac was used considering the OTU abundance along with the Adonis test of statistical significance (p<0.05), which is a non-parametric multivariate analysis of variance (MANOVA) with the Adonis function and utilizes the sample-to-sample distance matrix directly for finding significant differences among these communities [45].

## Results and Discussion

### Microbiological quality of fresh rainwater and reservoir water

Microbial quality is usually assessed by measuring ‘fecal indicator bacteria’ (also referred to as fecal indicator organisms, or FIOs) that are generally opportunistic pathogens, present in large numbers in fecal materials. Their presence in water samples is used to indicate the presence of fecal pollution and the possibility that fecal associated pathogens may also be present. The most commonly examined FIOs are thermotolerant coliforms (also termed fecal coliforms), *Escherichia coli* and intestinal *Enteroco*cc*i* (also termed fecal *streptococci*). *E. coli* are considered the best indicators of fecal contamination in water. The presence of thermotolerant coliforms/*E. coli* in water is unacceptable from the public health perspective as it indicates that a major health risk exists. However, it should be noted that certain members of the coliform group live outside of the gastrointestinal tract in the environment and may create a false indication of fecal contamination [46]. Also, several strains of *Escherichia coli* which are of environmental origin can be thermotolerant, thus giving false positive results [47]. It was therefore necessary to evaluate a range of relevant indicator organisms and total bacteria.

**Figure 2:**
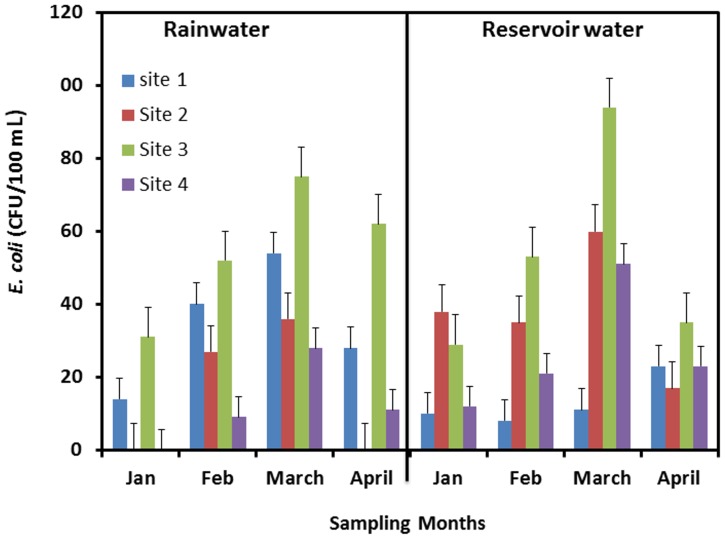
*E. coli* counts from both rainwater samples and reservoir samples collected from four sites from January to April, 2012.

**Figure 3:**
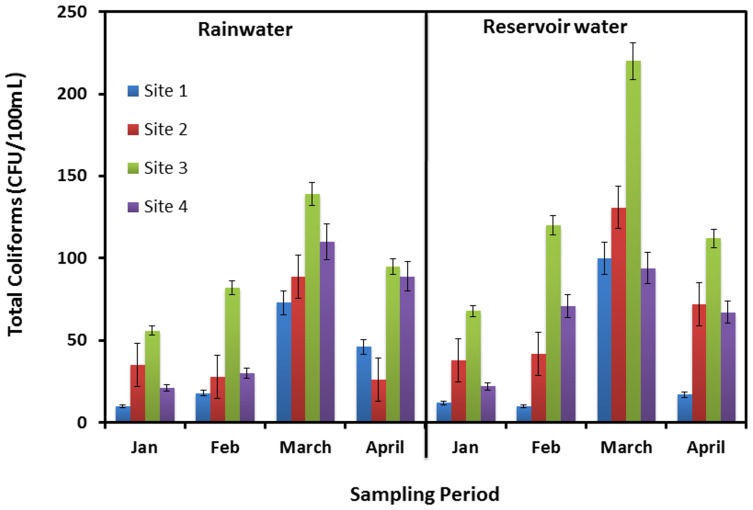
Total coliforms counts from both rainwater samples and reservoir samples collected at four sites from January to April, 2012.

**Figure 4:**
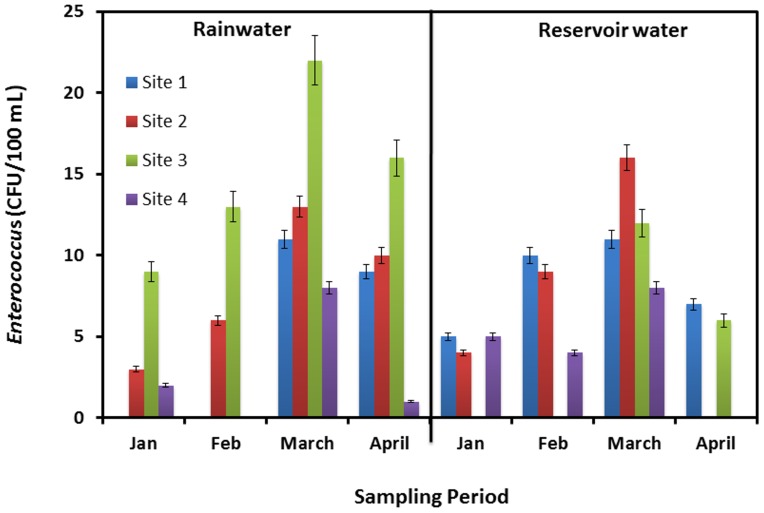
*Enterococcus* counts from both rainwater samples and reservoir samples collected at four sites from January to April, 2012.

Figs. 2, 3 and 4 show these microbial indicators for both fresh rainwater and reservoir water over the four months of sampling. Levels of thermotolerant coliforms/*E. coli* are expressed as colony forming units (CFU) per 100 mL (CFU/100 mL). The levels of *E. coli* were found to be in the range of 0 CFU/100 mL–75 CFU/100 mL for fresh rainwater and in the range of 10 CFU/100 mL–94 CFU/100 mL in reservoir water (Fig. 2). According to the WHO guidelines for treated drinking water (2006), the levels of *E. coli* should be less than 1 CFU/100 mL [48]. Thus, fresh rainwater is non potable for direct consumption and needs to be treated for potable purposes.

Total coliforms were previously considered indicators of fecal contamination. The NHMRC and AWRC “Guidelines for Drinking Water Quality in Australia” (2004) do not consider total coliforms as useful indicators of fecal contamination in the absence of *E. coli*, and have not proposed any guideline value for total coliforms [49]. As the case with HPC, total coliforms can also be used as an indicator for the effectiveness of any treatment program. However, it should be noted that while fecal coliforms are enteric organisms and therefore virtually exclusively of fecal origin, the total coliform count may comprise environmental organisms that are not necessarily common to the digestive tracts of vertebrate animals [50]. The total coliforms were found in the range of 10 CFU/100 mL–220 CFU/100 mL in reservoir water as compared to 10 CFU/100 mL–139 CFU/100 mL in fresh rainwater (Fig. 3). The highest counts were obtained from site #3 in both rainwater and reservoir water, which is near a major highway with residential buildings in the vicinity. This anthropogenic influence could be due to the abundance of airborne microbial pathogens in urban areas with high population density [1], [9]; Turkum *et al.* (2008) reported that most of the species found in rainwater were derived from aerosol and gas-phase components [51].

*Enterococci* are a specific group of bacteria that are found in high numbers in both human and animal faeces, and are therefore a valuable indicator for determining the extent of fecal contamination of a water source [16], [52]–[53]. *Enterococci* were found to be in the range of 0 CFU/100 mL–11 CFU/100 mL in fresh rainwater and 0 CFU/100 mL–35 CFU/100 mL in the reservoir water (Fig. 4). Overall, the four microbial indicators were found to be the highest at site #3 in both types of water samples. Thus, the results of our microbiological indicator analyses suggested that both the fresh rainwater and the reservoir water samples are not suitable for human consumption without any treatment similar to the findings on the roof harvested rainwater [22], [52], [54].

**Figure 5:**
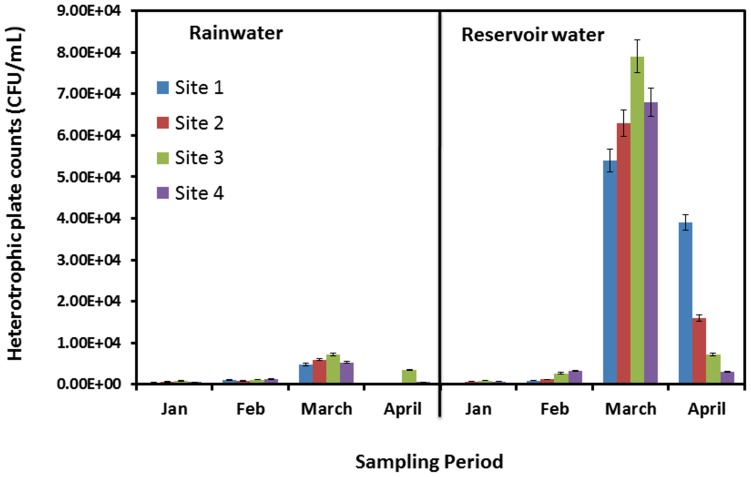
Total heterotrophic plate count bacteria from both rainwater samples and reservoir samples collected at four sites from January to April, 2012.

Total HPC bacteria of the reservoir water samples were two orders of magnitude higher than those of the rainwater. The baseline levels for the reservoir water ranged from 330 CFU/mL to 7.9×10^4^ CFU/mL, as compared to 280 CFU/mL to 7.2×10^2^ CFU/mL in fresh rainwater (Fig. 5). The reason for the reservoir water having two fold higher magnitudes of total HPC bacteria and slightly higher number of total coliforms could be the additional contribution from sediments apart from rainfall and riverine inflows. HPC bacteria are carried by runoff into the reservoir from highland agricultural areas during intensive rainfall events. Thus, rain events may lead to an inflow of high nutrient concentrations as well as high loads of microbes [55]–[56]. Based on the microbial indicator numbers, there was no statistically significant difference between the rainwater and reservoir water quality with respect to *E. coli* and *Enterococci*. However, the microbial quality of rainwater and reservoir was found to be significantly different (p≤0.05) for total heterophic counts and total coliforms according to the t-test. It was also observed that the concentrations of all the microbial indicators were higher in the month of March as compared to those in other months. This can be attributed to a higher frequency of rainfall in March as compared to the relatively dry period in the months of January and February.

It should be noted that *E. coli and Enterococci* are indicative of recent fecal contamination as compared to heterophic counts and total coliforms. The influence of *E. coli and Enterococci* of atmospheric origin on the reservoir water quality appears to be relatively less as compared to that of total bacteria HPC and total coliforms, this could due to their lower abundance in the atmosphere and/or the influence of physical processes such as advection, dispersion, diffusion resulting in low residence time [22]. Other processes such as competitive exclusion and nutrient change may also play role to regulate the survival of these incoming bacteria [5].

### Microbial composition and Phylogenetic analyses of fresh rainwater and reservoir water

Microbial communities are fundamental to the functioning of aquatic ecosystems. With the availability of metagenomic technologies and approaches [57], it is important to know the changes in total microbial diversity of both rainwater and reservoir water in addition to the changes in microbial indicator numbers. Gaining insights into the total microbial community would provide a better understanding of its role in mediating aquatic ecological processes and biogeochemical cycling [57]–[58]. However, the roles of most microorganisms in natural systems are unclear as most of them cannot be cultivated for investigation by current conventional culturing methods [59]. The introduction of culture-independent molecular methods has shed light on the determination of community compositions, and laid the foundation for gaining a deep understanding of aquatic microbial ecology. Rainwater has received considerable attention as a potential alternative source of potable and non-potable water in regions where there is water scarcity [60]. Rainwater collection is an ancient practice and currently still being practiced, especially in areas with no running water. However, the use of 16 S rRNA sequencing techniques has not been applied to fresh rainwater investigations. Thus, there is a lack of information on its microbial communities and their role in aquatic microbial ecology.

**Figure 6:**
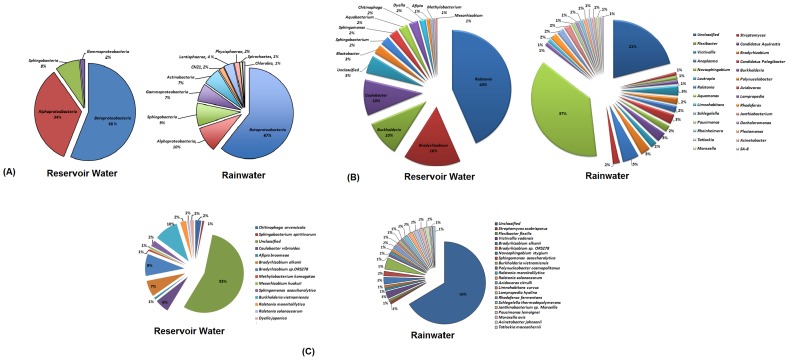
Bacteria taxon richness on (A) class level (B) genus level and (C) species level observed in the fresh rainwater and reservoir water.

In this study, 10 classes of bacteria were detected in fresh rainwater and four classes of bacteria in reservoir water. In fresh rainwater, sequences were affiliated with *Betaproteobacteria, Alphaproteobacteria, Sphingobacteria, Actinobacteria, Gammaproteobacteria, Lentisphaerae, CH21, Phycisphaerae, Chlorobia* and *Spirochaetes*. In contrast, the reservoir water library detected sequences affiliated with only *Betaproteobacteria, Alphaproteobacteria, Sphingobacteria* and *Gammaproteobacteria. Betaproteobacteria* was found to be the dominant class in both the libraries (Fig. 6). The fresh rainwater had higher diversity and taxonomic richness at the class level than those of reservoir water as the numbers of OTUs were found to be 63 and 29 for fresh rainwater and reservoir water, respectively. However, the proportion of *Alphaproteobacteria* in the bacterial community was found to be higher in the reservoir water than in the fresh rainwater. *Alphaproteobacteria*, at least at the class level, are resistant to predation: their relative higher abundance in reservoir water may be due to the capacity to degrade recalcitrant organic compounds such as humic substances accompanied by a tendency of members of the *Alphaproteobacteria* to form filaments, aggregates, or *Caulobacter*-like stalked cells that sometimes can make up a majority of the *Alphaproteobacteria* population in freshwater lakes [61].

The taxonomic richness observed in both types of water samples and their relative distribution appears to be similar to that observed in many freshwater and marine systems, as determined by both cultivation and molecular techniques [22], [62]–[63]; these findings are consistent with the results from previous studies where the dominance of *Betaproteobacteria* has been observed consistently in freshwater systems, particularly among free-living groups, while α and γ sub-classes appear to dominate among particle-attached groups [64]. The high abundance and amenability to culturing have contributed to the *Betaproteobacteria* being the best-studied group in freshwater lakes [61]. Venter *et al.* (2004) carried out a comprehensive genomic study of natural waters at an oligotrophic ocean site in the Sargasso Sea, and found an abundant bacterial distribution dominated by *Proteobacteria* of α, β and γ sub-classes, followed by *Actinobacteria* and *Bacteroidetes* [65]. These findings are similar to those from the fresh rainwater microbial community analyses in the current study as well as from the study conducted by Evans *et al*., 2009 in rainwater tanks [22]. The class *Betaproteobacteria* was the dominant and the most diverse group in freshwater mesocosm and this dominance is suggested to be associated with their ability to respond quickly to nutrient additions [66]–[67]. The predominance of *Proteobacteria* in fresh rainwater can also be suggestive of the generally clean oligotrophic nature of fresh rainwater.

*Actinobacteria* are Gram-positive bacteria representing the most abundant group (50–70%) of total bacteria in various freshwater habitats [68]–[69] were also found to be present in fresh rainwater. Previous studies have found that members of *Actinobacteria* contributed to glucose assimilation and heterotrophic nitrification [70]–[71] and played a key role in nutrient and energy cycling in aquatic habits [71]. Also most interestingly, the UV stress resistance has also been postulated to be one of the reasons for the success of the *Actinobacteria* in the upper waters of lakes, which often have high UV transparency. Warnecke and colleagues showed a significant positive relationship between *Actinobacteria* abundance and UV transparency in study on mountain lakes [68]. In addition to UV protection, many members of the *Actinobacteria* are capable of producing spores, allowing them to survive long periods of desiccation. Thus, strong UV protection and desiccation resistance via spore formation, together with the known small cell size of these organisms, would make the freshwater *Actinobacteria* particularly suitable for aerial dispersal, thereby explaining their ubiquitous representation in globally dispersed lakes. However, to date, these taxa have rarely been identified in any air samples [61]. This is the first study to-date to detect their presence in fresh rainwater suggesting their ability to survive in airborne particles that tend to be scavenged by rainwater during rain events [1]. This wet-deposition process enables the global distribution of the microorganisms of atmospheric origin in aquatic ecosystems [66].

**Figure 7:**
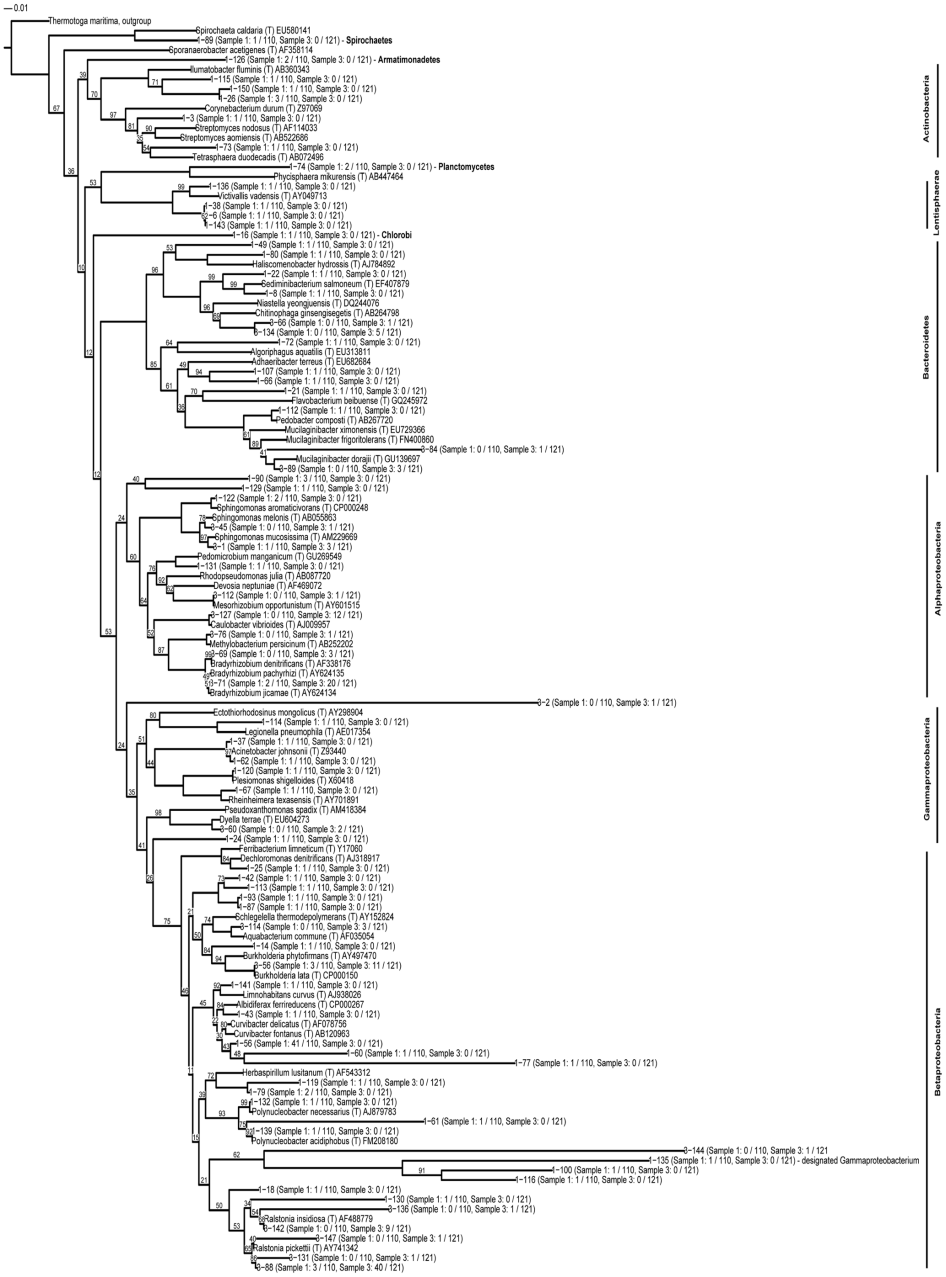
Taxonomic tree indicating the phylogenetic distribution of all bacterial groups in both rainwater and reservoir water samples.

Phylogenetic tree-based microbiome comparison of the fresh rainwater and reservoir water samples was performed by the neighbor-joining method of experimentally observed OTUs and reference type strains with each leaf representing an OTU displays counts of sequences observed in each sample (Fig. 7). Notable differences in the frequencies of the some of the bacterial genera were observed. *Bradyrhizobium, Chitinophaga, Caulobacter* and *Ralstonia* were found to be more abundant in reservoir water than in fresh rainwater whereas *Curvibacter* was found to be more abundant in fresh rainwater than in reservoir water. However, unlike taxonomic richness, the coverage of the fresh rainwater clone library (150 sequences from pooled DNA) as calculated based on the species OTU (97% 16 S rRNA gene sequence identity) by Good's Clone Coverage [42] was low (77%) as compared to the coverage of the reservoir water clone library (92%).

The OTU with the greatest difference in relative abundance was associated with *Curvibacter* for fresh rainwater and with *Ralstonia* for reservoir water. *Curvibacter* and *Ralstonia* belong to the *Burkholderiales* order of *Betaproteobacteria*, under the taxa Comamonadaceae that is reported to be the most abundant typical freshwater fast growing and nutrient-loving group [31], [72]. Rainwater and reservoir samples formed separate groups in ordination analysis, but were not found to be significantly dissimilar (Adonis p-value  = 0.125) using OTU abundance metrics. The same diversity of bacteria being present in two different environmental samples suggests that the microbiological composition of the atmosphere and that of the hydrosphere are intimately linked with each other through wet and dry deposition mechanisms. The type of bacterial diversity present in fresh rainwater, the general abundance distribution, and the resemblance of the composition to that of reservoir, have indicated the likely existence of definable micro ecosystems in fresh rainwater and their impact on other aquatic systems. The functional operation of a stable micro-ecology, dominated by well-adapted core resident groups, may have implications with regard to the harvesting of rainwater and for management of water resources.

## Conclusions

This study focused on the investigation of the microbial diversity of fresh rainwater and that of reservoir water with a concurrent assessment of bacterial counts and species profiles in a tropical region, characterized by abundant rainfall throughout the year, for the first time. The results of the study suggested that despite the presence of bacterial pathogens in rainwater and their wet deposition through rainfall, there was no significant increase in the microbial loading of the tropical reservoir water. It appears that the incoming bacterial loads from the rainwater entering the reservoir either undergo dilution, or are not sustained for a long period of time due to the influence of physical processes such as advection, dispersion and diffusion and other processes (competitive exclusion and nutrient change) that may act to regulate the survival of incoming bacteria. Thus, despite the presence of microbial pathogens, rainwater harvesting in large water catchment areas is a promising freshwater resource following treatment. These findings on the composition of aquatic microbial communities in the reservoir water and fresh rainwater by 16 s rRNA clone libraries indicated that the members of *Betaproteobacteria* dominated both the communities. The OTU with the greatest difference in the relative abundance for fresh rainwater was *Curvibacter* whereas for reservoir water it was *Ralstonia*. *Actinobacter* was detected in fresh rainwater, suggesting their presence in airborne particles as well. The fresh rainwater showed greater taxonomic richness than that of the reservoir water.

However, due to lower coverage in traditional cloning method, our understanding about the bacterial composition and phylogenetic diversity of the bacterial community in the freshwater is still limited. Hence, high-throughput molecular tools such as pyrosequencing and hybridization to PhyloChip array that are able to elucidate not only cultivable bacteria, but also viable-but-not-cultivable (VBNC) may extend and expand our understanding of the tropical freshwater reservoir microbial communities. More in-depth studies on the linkage between atmospheric inputs of microorganisms and the microbial diversity of freshwater resources are warranted. This research group has recently performed study of reservoir microbial diversity using PhyloChip microarrays at the same reservoir in the presence and absence of rain events (work in progress), which may add more insights into differences between the traditional cloning and metagenomics of freshwater bacterial diversity.

## Supporting Information

Table S1 **Summary of physico chemical analyses of Fresh rainwater and reservoir water quality.**
(DOCX)

Text S1 **Physico-Chemical Analyses.**
(DOC)
