# Supplementary material for: Microbial Quality and Phylogenetic Diversity of Fresh Rainwater and Tropical Freshwater Reservoir
Source: PLoS One. 2014 Jun 30;9(6):e100737. doi: 10.1371/journal.pone.0100737 (PMC4076214; doi:10.1371/journal.pone.0100737)
Supplement: Table S1 — Summary of physico chemical analyses of Fresh rainwater and reservoir water quality. (DOCX) [file pone.0100737.s001.docx]

**Table S1.** Summary of physico chemical analyses of Fresh rainwater and reservoir water quality

| **Parameter** | **Result for:** | |
| --- | --- | --- |
|  | **Rainwater** | **Reservoir water** |
| **pH** | 4.2 | 7.2 |
| **Turbidity (NTU)** | 0.91 | 3.56 |
| **Ammonium (mg/liter)** | 0.52 | 0.15 |
| **Nitrite (mg/liter)** | 0.04 | 0.16 |
| **Nitrate (mg/liter)** | 0.90 | 1.58 |
| **Phosphate (mg/liter)** | 0.54 | 0.34 |
| **Conductivity (μS/cm)** | 25.33 | 267.22 |

Hot Spots
