# Supplementary material for: Microbial Quality and Phylogenetic Diversity of Fresh Rainwater and Tropical Freshwater Reservoir
Source: PLoS One. 2014 Jun 30;9(6):e100737. doi: 10.1371/journal.pone.0100737 (PMC4076214; doi:10.1371/journal.pone.0100737)
Supplement: Text S1 — Physico-Chemical Analyses. (DOC) [file pone.0100737.s002.doc]

**Text S1**

***Physico-Chemical Analyses***

For the chemical analyses, pH, conductivity, turbidity and conductivity were measured for all the rainwater and reservoir water samples. All these parameters were analyzed immediately upon arrival at the laboratory. The pH was measured with a combination electrode and meter. Conductivity and turbidity were measured with a conductivity meter and turbidity meter. Samples for, ammonia, phosphorous, nitrate and nitrite analysis were stored at 4^o^C and analyzed within 48 hrs. The samples were tested for the Ammonia, Nitrate, Nitrite and phosphorus content according to Standard Methods (APHA, 1998).

**Reference:**

American Public Health Association (APHA). Standard Methods for Examination of wastewater. 20th ed., American Public Health Association, American Water Works Association, Water Environmental Federation, Washington, DC.1998.
